# Pharmacokinetics and Tolerance of the Phage Endolysin-Based Candidate Drug SAL200 after a Single Intravenous Administration among Healthy Volunteers

**DOI:** 10.1128/AAC.02629-16

**Published:** 2017-05-24

**Authors:** Soo Youn Jun, In Jin Jang, Seonghae Yoon, Kyungho Jang, Kyung-Sang Yu, Joo Youn Cho, Moon-Woo Seong, Gi Mo Jung, Seong Jun Yoon, Sang Hyeon Kang

**Affiliations:** a iNtRON Biotechnology, Inc., Seongnam-si, Gyeonggi-do, Republic of Korea; b Department of Clinical Pharmacology and Therapeutics, Seoul National University Hospital, Seoul, Republic of Korea; c Department of Laboratory Medicine, Seoul National University Hospital, Seoul, Republic of Korea

**Keywords:** phage endolysin, SAL200, phase 1 clinical study, staphylococcal infections

## Abstract

This study was a phase 1, single-center, randomized, double-blind, placebo-controlled, single-dosing, and dose-escalating study of intravenous SAL200. It is a new candidate drug for the treatment of antibiotic-resistant staphylococcal infections based on a recombinant form of the phage endolysin SAL-1. The study evaluated the pharmacokinetics, pharmacodynamics, and tolerance among healthy male volunteers after the intravenous infusion of single ascending doses of SAL200 (0.1, 0.3, 1, 3, and 10 mg/kg of body weight). SAL200 was well tolerated, and no serious adverse events (AEs) were observed in this clinical study. Most AEs were mild, self-limiting, and transient. The AEs reported in more than three participants were fatigue, rigors, headache, and myalgia. No clinically significant values with respect to the findings of clinical chemistry, hematology, and coagulation analyses, urinalysis, vital signs, and physical examinations were observed, and no notable trends in our electrocardiogram (ECG) results for any tested dose were noticed. A greater-than-dose-proportional increase with regard to systemic exposure and the maximum serum concentration was observed when the SAL200 dose was increased from 0.1 mg/kg to 10 mg/kg. This investigation constitutes the first-in-human phase 1 study of an intravenously administered, phage endolysin-based drug. (This study has been registered at ClinicalTrials.gov under identifier NCT01855048 and at the Clinical Research Information Service [https://cris.nih.go.kr/cris/] under identifier KCT0000968.).

## INTRODUCTION

A wide variety of bacteria can cause severe infections. Many of these bacteria are or have become resistant to many commonly used antibiotics. Infections due to drug-resistant bacteria require treatment with new types of antibiotics. Multidrug-resistant bacteria cause an increasing number of deadly infections. The Centers for Disease Control and Prevention (CDC) estimated that approximately 94,360 people in the United States developed a serious methicillin-resistant Staphylococcus aureus (MRSA) infection in 2005. Of note, approximately 18,650 people died during hospitalizations related to these serious MRSA infections (1). For many years, MRSA remained a problem that was restricted to hospitals, intensive care units, and other medical health care facilities. The use of last-resort antibiotics, such as vancomycin, is necessary for treating systemic MRSA infections (2, 3). However, increasing evidence suggests that vancomycin is losing its clinical and microbiological potency, thereby resulting in an increased demand for novel antibiotics (4–6).

Since only a few novel antibiotics currently exist, phage endolysins are being more frequently considered potential treatments for bacterial infections. Phage endolysins, also known as phage lysins or lysins, are bacteriophage-encoded, peptidoglycan-degrading enzymes that rapidly degrade bacterial cell walls and release phage progeny (7). The exogenous application of purified recombinant phage endolysin proteins to Gram-positive bacteria induces rapid lysis and bacterial cell death (8–10). Since their discovery, phage endolysins were proposed to be antibacterial agents because of their distinct mode of action and highly specific antibacterial activity, which are independent of bacterial antibiotic susceptibility patterns (9). Several previous studies have applied phage endolysins to target pathogens such as Bacillus anthracis (11), Streptococcus pneumoniae (12), Staphylococcus aureus (13), and Bacillus thuringiensis (14), and promising results were obtained using animal models of human disease (12, 15–22). Phage endolysins differ from standard-of-care antibiotics with regard to their potency, speed of bactericidal activity, specificity, and activity against antibiotic-resistant strains. In addition, phage endolysins are generally highly specific for particular bacterial species and rarely lyse nontarget bacteria, including commensal bacteria, which might reduce clinical complications. Therefore, phage endolysins represent a promising research target for the discovery and development of novel antibacterial therapeutic agents (23).

SAL200 is a new phage endolysin-based candidate drug used to treat staphylococcal infections. SAL200 is formulated for injection, and it contains a recombinant form of phage endolysin SAL-1 (rSAL-1) as its active pharmaceutical ingredient (24, 25). rSAL-1 is derived from the bacteriophage SAP-1, which infects staphylococci, including MRSA and vancomycin-resistant S. aureus (VRSA) strains. Early studies demonstrated the potency of SAL200 as an antistaphylococcal agent (24, 25). Our previous studies showed that rSAL-1 exhibited rapid and effective bactericidal activity against encapsulated and biofilm-forming S. aureus cells and also against planktonic S. aureus cells. rSAL-1 also displayed broad-spectrum lytic activity against S. aureus isolates, including MRSA strains. Furthermore, the intravenous injection of SAL200 into a mouse model of MRSA infection prolonged the viability of the mice and significantly reduced bacterial counts in the bloodstream and the spleen. The rapid and effective bactericidal activity of SAL200 suggests that a brief SAL200 dosing period is sufficient to treat staphylococcal infections. In addition, favorable safety evaluation results were obtained in good laboratory practice (GLP)-compliant studies that evaluated the safety of intravenously administered SAL200 in rats (26), dogs (26), and monkeys (27).

The aim of this first-in-human phase 1 study was to assess the safety, tolerability, pharmacokinetics, and pharmacodynamics of SAL200 among healthy adults after the infusion of single ascending intravenous doses.

## RESULTS

### Demographics.

**FIG 1:**
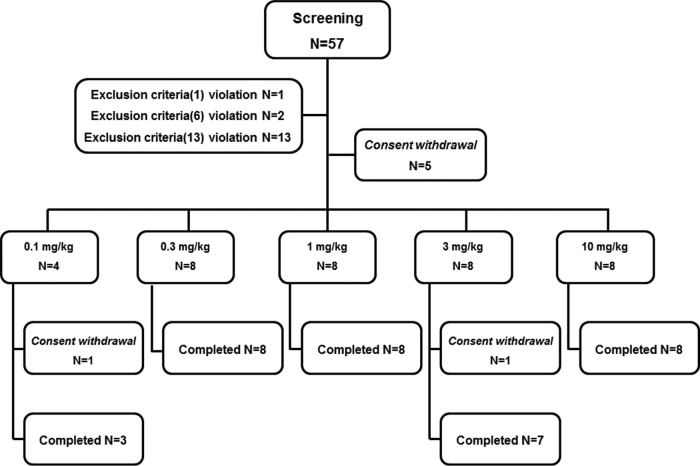
Consolidated Standards of Reporting Trials (CONSORT) diagram. Exclusion criteria (1), subjects with evidence or history of clinically significant hepatic, renal, neurologic, endocrine, pulmonary, hematological, neoplastic, cardiovascular, or psychiatric disease. Exclusion criteria (6), subjects who have systolic blood pressure <90 mmHg or diastolic blood pressure <50 mmHg or systolic blood pressure >150 mmHg or diastolic blood pressure >100 mmHg when measured at a sitting position after resting for 3 min. Exclusion criteria (13), subjects who are evaluated as ineligible for study participation for other reasons, including clinical laboratory test results.

A total of 57 men volunteered for screening. All volunteers were Korean men. Of these volunteers, 36 were enrolled. The remaining 21 were excluded from the study because 5 of these participants did not provide informed consent, 9 participants were removed from the analysis because of abnormal clinical findings, 1 participant was removed because of abnormal electrocardiogram (ECG) findings, 2 participants were removed because of abnormal vital signs, 1 participant was removed because of caffeine consumption, 1 participant was removed because of an unsuitable medical history, and 2 participants withdrew because of abnormal skin tests. Regarding the two volunteers with abnormal skin test results, one was excluded because of a skin test abnormality showing a positive response on his right arm (which was treated with placebo) but a negative response on his left arm (which was treated with SAL200), whereas the second volunteer showed a negative response on his right arm (which was treated with placebo) and a positive response on his left arm (which was treated with SAL200). All 36 volunteers completed dosing and testing at the 7-day follow-up visit, and 2 participants (participants R102 and R406) withdrew after test completion at the 7-day follow-up visit. Thus, in total, 34 volunteers completed this clinical study (Fig. 1). The demographic data for the 36 enrolled participants are summarized in Table 1.

**TABLE 1:** Demographic and baseline characteristics

Characteristic	Value(s) for the following group (dose [mg/kg]):					
	Cohort 1 (0.1)	Cohort 2 (0.3)	Cohort 3 (1)	Cohort 4 (3)	Cohort 5 (10)	Total
No. of subjects receiving:						
Active pharmaceutical ingredient	3	6	6	6	6	27
Placebo	1	2	2	2	2	9
Median (range) age (yr)	25 (23–26)	27.5 (23–33)	27.5 (23–39)	23.5 (20–29)	29 (24–38)	27 (20–39)
Mean (SD) wt (kg)	68.3 (4.4)	65.2 (8.7)	73.4 (4.8)	68.7 (5.6)	67.6 (3.7)	68.7 (6.2)
Mean (SD) ht (cm)	176.9 (4.2)	171.1 (3.3)	175.8 (3.6)	174.4 (2.2)	171.7 (5.0)	173.6 (4.1)
Mean (SD) BMI^*a*^ (kg/m^2^)	21.7 (2.5)	22.2 (2.5)	23.8 (2.1)	22.0 (2.0)	23.0 (1.5)	22.8 (2.1)

a BMI, body mass index.

### Safety and tolerance.

We evaluated the safety and tolerance of single ascending, intravenously infused doses of SAL200 in healthy men who were randomly assigned to receive either SAL200 or placebo across five cohorts (cohorts receiving SAL200 at 0.1, 0.3, 1, 3, and 10 mg/kg of body weight). Dosing was conducted between August and November 2013, and the 50-day poststudy visit tests were completed in January 2014.

No serious adverse events (AEs) were observed throughout the study. All observed AEs are shown in Table 2. A total of 42 AEs were reported for 12 of the 36 participants across all dose groups. Nine participants exhibited 32 AEs, which were possibly related to either SAL200 or the placebo, and 7 participants presented 10 AEs that were considered unrelated to either SAL200 or the placebo. The AEs that were possibly related to medicinal products included 30 mild AEs and 2 moderate AEs. Of these AEs, one case of headache was reported in a placebo-treated participant. Bilirubinemia (the presence of increased amounts of bilirubin in the blood) was reported in one participant (R208), as determined during clinical laboratory testing on day 2. His bilirubin level was determined to be 4.0 mg/dl. The bilirubinemia reported in participant R208 was considered to be unrelated to the medicinal product on the following basis: first, his bilirubin level was unstable prior to dosing. At his screening visit, his bilirubin level was 3.4 mg/dl in the first clinical laboratory test and 1.7 mg/dl when the test was repeated. This indicated that his bilirubin level was unstable. Although his bilirubin level was unstable, he was enrolled because his bilirubin level on the repeat test was within the normal range. Second, his bilirubinemia disappeared without medical treatment when it was tested on the following day. Third, no abnormalities in his vital sign measurements, in his ECG measurements, or during his physical examination after dosing were detected.

**TABLE 2:** All AEs observed after a single intravenous dose among healthy male volunteers (group summary)

Observed AE	Value for the following group (dose [mg/kg])^*a*^:													
	Placebo		Cohort 1 (0.1)		Cohort 2 (0.3)		Cohort 3 (1)		Cohort 4 (3)		Cohort 5 (10)		Total	
	R	U	R	U	R	U	R	U	R	U	R	U	R	U
General disorders and administration site conditions														
Fatigue											3 (3)		3 (3)	
Fever											1 (1)		1 (1)	
Application site pain						1 (1)								1 (1)
Rigors					1 (1)						4 (4)		5 (5)	
Respiratory, thoracic, and mediastinal disorders														
Coughing							1 (1)					1 (1)	1 (1)	1 (1)
Pharyngitis							1 (1)			1 (1)	1 (1)		2 (2)	1 (1)
Rhinitis							1 (1)				2 (2)	2 (2)	3 (3)	2 (2)
Nervous system disorders														
Headache	2 (1)	1 (1)									3 (3)		5 (4)	1 (1)
Dizziness											2 (2)		2 (2)	
Syncope						1 (1)								1 (1)
Gastrointestinal disorders														
Abdominal pain										1 (1)	1 (1)		1 (1)	1 (1)
Diarrhea										1 (1)				1 (1)
Nausea											2 (2)		2 (2)	
Dyspepsia											1 (1)		1 (1)	
Musculoskeletal and connective tissue disorders														
Back pain											1 (1)		1 (1)	
Myalgia					1 (1)						3 (2)		4 (3)	
Vascular disorders, hypotension											1 (1)		1 (1)	
Hepatobiliary disorders, bilirubinemia						1 (1)								1 (1)

a Data are presented as the number of adverse events (number of participants with adverse events). R, related (probable/likely and possible) to the medicinal product; U, unrelated (unlikely and not related) to the medicinal product.

Most AEs were mild, self-limiting, and transient. Drug therapy for AEs was administered for only five participants (participants R501, R502, R503, R505, and R508), and these participants were in cohort 5, the group receiving the highest dose (10 mg/kg). Concomitant acetaminophen was administered to these five participants, and their symptoms resolved without further effects. Most of the AEs were observed in three participants in cohort 5. The AEs observed in all participants in cohort 5 along with the dosing dates are presented in Table S1 in the supplemental material. Dosing with the 10-mg/kg dose was conducted over 3 days. For AEs that were observed on day 1 (i.e., the dosing day) and considered to be related to either SAL200 or placebo, 16 AEs were observed in three participants on the same dosing day; the other 4 AEs were observed in three participants (3 AEs in 2 active pharmaceutical ingredient-treated volunteers and 1 AE in 1 a placebo-treated volunteer) on different dosing days.

No risks were identified after evaluation of the vital signs, ECG, and physical measurements of the participants, and no clinically remarkable changes in serum chemistry, hematology, or urinalysis test results were observed throughout the study. All principal hematologic parameters (i.e., hematocrit, platelet, and leukocyte counts), coagulation parameters (i.e., prothrombin and activated partial thromboplastin times), and serum chemistries (i.e., alanine aminotransferase and aspartate aminotransferase levels) were within the normal ranges.

### Immunogenicity.

To assess the humoral immune response to SAL200, the levels of antibodies against rSAL-1 (anti-rSAL-1 antibodies) in serum samples collected at the 50-day poststudy visit were determined. The serum levels of the anti-rSAL-1 antibodies are presented in Table 3. Anti-rSAL-1 antibodies were detected in 10 of 27 active pharmaceutical ingredient-treated participants (37%), most of whom belonged to cohorts 3 to 5. Although the levels of anti-rSAL-1 antibodies showed relatively large variations among samples, they ranged from 2 to 5 μg/ml. The highest serum level of anti-rSAL-1 antibodies was 12.055 μg/ml, which was determined in one volunteer in cohort 4. Regarding the relationship between the anti-rSAL-1 antibody level and dose, the anti-rSAL-1 antibody level appeared to be proportional to the dose, although the available data were limited. However, the level for cohort 5 was relatively lower than that for cohort 4 (Table 4).

**TABLE 3:** Serum levels of anti-rSAL-1 antibodies determined at the 50-day poststudy visit in each participant

Participant identification code	Treatment	Dose (mg/kg)	Antibody level^*a*^ (μg/ml)
R101	Active^*b*^	0.1	NQ
R102	Active	0.1	NA
R103	Placebo	0.1	NQ
R104	Active	0.1	NQ
R201	Active	0.3	NQ
R202	Active	0.3	NQ
R203	Active	0.3	2.833
R204	Placebo	0.3	NQ
R205	Active	0.3	NQ
R206	Placebo	0.3	NQ
R207	Active	0.3	NQ
R208	Active	0.3	NQ
R301	Placebo	1	NQ
R302	Active	1	NQ
R303	Active	1	NQ
R304	Active	1	NQ
R305	Placebo	1	NQ
R306	Active	1	1.916
R307	Active	1	3.562
R308	Active	1	2.035
R401	Active	3	12.055
R402	Active	3	NQ
R403	Placebo	3	NQ
R404	Active	3	NQ
R405	Active	3	5.326
R406	Active	3	NA
R407	Placebo	3	NQ
R408	Active	3	3.759
R501	Active	10	NQ
R502	Active	10	9.990
R503	Active	10	NQ
R504	Placebo	10	NQ
R505	Active	10	4.684
R506	Active	10	NQ
R507	Placebo	10	NQ
R508	Active	10	5.068

a NQ, not quantifiable as the value was less than the LLOQ (1.563 μg/ml); NA, not assayed, as a sample was unavailable.

b Active, active pharmaceutical ingredient.

**TABLE 4:** Serum levels of anti-rSAL-1 antibodies according to SAL200 dose

Dose (mg/kg)	Serum level (μg/ml) of anti-rSAL-1 antibody (no. of participants)^*a*^
0.1	0
0.3	2.833 (1)
1.0	2.50 ± 0.92 (3)
3.0	7.05 ± 4.41 (3)
10.0	6.58 ± 2.96 (3)

a All data are presented as the mean ± SD.

### Pharmacokinetics.

**FIG 2:**
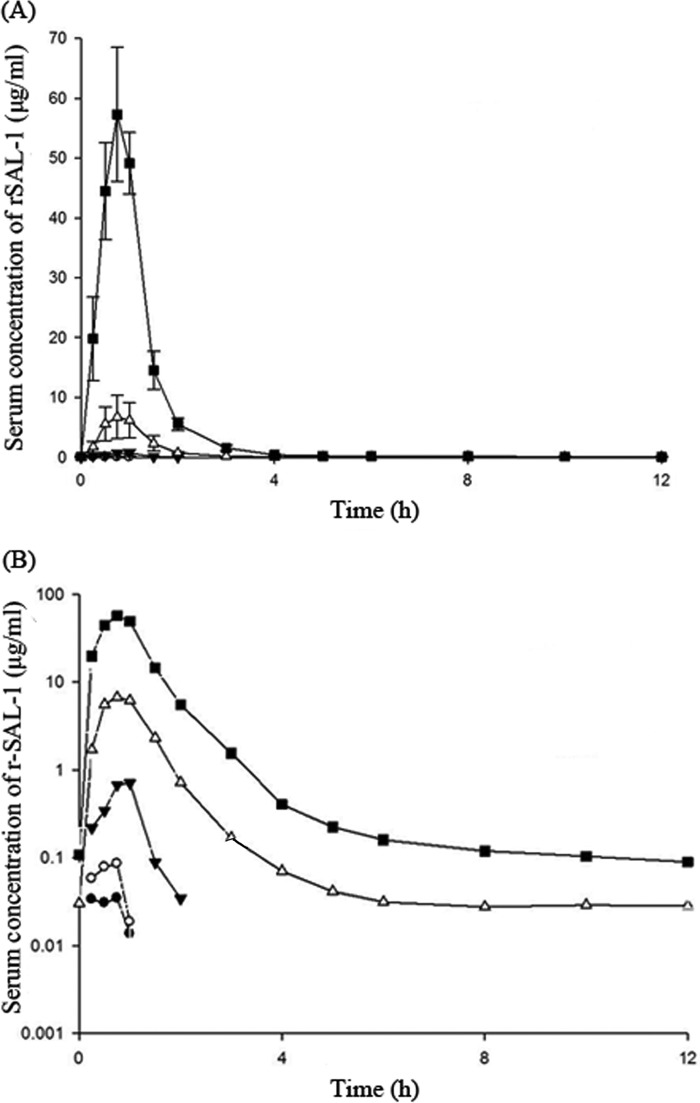
Mean single-dose serum concentration-time profiles of SAL200. Linear (A) and log-transformed (B) profiles are shown. •, 0.1 mg/kg; ○, 0.3 mg/kg; ▼, 1 mg/kg; △, 3 mg/kg; ■, 10 mg/kg. Error bars denote SDs.

Pharmacokinetics were evaluated by measuring the serum concentrations of SAL200 at specified times after administration, and these concentrations were used to determine the pharmacokinetic parameters maximum drug concentration in serum (*C*_max_), the time to *C*_max_ (*T*_max_), effective half-life (*t*_1/2_), and the area under the serum concentration-time curve (AUC). A summary of the values for the pharmacokinetic parameters following a single intravenously infused dose of SAL200 is presented in Fig. 2 and Table 5. Following a 1-h intravenous infusion, the serum levels of SAL200 rapidly peaked, with the *T*_max_ values across the 0.1-, 0.3-, 1-, 3-, and 10-mg/kg doses being similar and ranging from 0.25 to 1.02 h. A greater-than-dose-proportional increase in *C*_max_ was observed. Following the attainment of *C*_max_, the serum concentration of SAL200 decreased biphasically. The effective mean terminal *t*_1/2_ ranged from 0.04 to 0.38 h. The AUCs, i.e., the area under the serum concentration-time curve from time zero to the time at which the last sample with a measurable concentration (a concentration above the lower limit of quantification [LLOQ]) was obtained (AUC_last_) and the area under the serum concentration-time curve up to the last time point with a concentration above the LLOQ extrapolated to infinity (AUC_inf_), increased in magnitude as the dose increased. The AUC increased in a greater-than-dose-proportional manner when the dose was increased from 0.1 mg/kg to 10 mg/kg.

**TABLE 5:** Values of pharmacokinetic parameters for SAL200 after a single intravenous dose among healthy male volunteers^*a*^

Dose (mg/kg)	*C*_max_ (μg/ml)^*b*^	*T*_max_ (h)^*c*^	Effective *t*_1/2_ (h)^*b*^	AUC_last_ (μg · h/ml)^*b*^	AUC_inf_ (μg · h/ml)^*b*^
0.1 (*n* = 3)	0.04 ± 0.01	0.25 (0.25–0.75)	0.04 ± 0.02	0.03 ± 0.005	
0.3 (*n* = 6)	0.09 ± 0.04	0.63 (0.5–0.75)	0.04 ± 0.01	0.05 ± 0.02	
1 (*n* = 6)	0.82 ± 0.50	0.75 (0.5–1.0)	0.25 ± 0.05	0.57 ± 0.31	0.66 ± 0.30
3 (*n* = 6)	7.10 ± 3.59	0.88 (0.75–1.0)	0.38 ± 0.06	7.26 ± 3.42	7.28 ± 3.43
10 (*n* = 6)	57.99 ± 10.37	0.75 (0.75–1.02)	0.38 ± 0.05	59.79 ± 9.03	59.86 ± 9.04

a *C*_max_, maximum drug concentration in serum; *T*_max_, time to *C*_max_; *t*_1/2_, terminal half-life over the sampling period; AUC_last_, area under the serum concentration-time curve from time zero to the time at which the last sample with a measurable concentration was obtained; AUC_inf_, area under the serum concentration-time curve up to the last time point with a concentration above the LLOQ extrapolated to infinity.

b Data are presented as the mean ± SD.

c Data are presented as the median (range).

### Pharmacodynamics.

Pharmacodynamics were measured using an *ex vivo* blood assay for determining blood antibacterial activity. The blood antibacterial activity was assessed via a qualitative comparison of the clear zones (i.e., lysis halos) formed after spotting of the tested blood samples onto a lawn of S. aureus bacteria with those formed after the spotting of standards. Blood antibacterial activity was detected in all blood samples collected from active pharmaceutical ingredient-treated participants in cohorts 3 to 5 at 1 h after dosing. The antibacterial activity in the blood samples collected from participants in cohorts 1 and 2 was below the detection limit of the assay (0.05 μg/ml). Blood antibacterial activity was detected in some of the blood samples collected from participants in cohort 5 at 2 h after dosing. The pharmacodynamic assay results are presented in Table 6. The blood antibacterial activity after the intravenous administration of a single dose of SAL200 was approximately proportional to the dose. Blood antibacterial activity of greater than 0.1 μg/ml was detected in all blood samples collected from all active pharmaceutical ingredient-treated participants in cohorts 3 to 5 at 1 h after dosing.

**TABLE 6:** Results of blood antibacterial activity assay

Dose (mg/kg)	Pharmacodynamic assay result (μg/ml)^*a*^		
	1 h	1.5 h	2 h
0.1 (*n* = 3)	NQ	NQ	NQ
0.3 (*n* = 6)	NQ	NQ	NQ
1 (*n* = 6)	0.13 ± 0.05 (6)	NQ	NQ
3 (*n* = 6)	0.22 ± 0.10 (6)	NQ	NQ
10 (*n* = 6)	0.40 ± 0.13 (6)	0.15 ± 0.05 (6)	0.08 ± 0.03 (3)

a All data are presented as the mean ± SD concentration (in micrograms per milliliter), and the number of participants is presented in parentheses. NQ, not quantifiable as the value was less than the LLOQ (0.05 μg/ml).

### Pharmacokinetic and pharmacodynamic analyses.

The relationship between the blood antibacterial activity and the serum concentration of SAL200 was analyzed by comparing the rSAL-1 serum level and the simultaneously measured blood antibacterial activity in the same participants. The blood antibacterial activity was approximately 100-fold lower than the corresponding serum concentration of SAL200, although blood samples with a similar serum concentration of SAL200 showed different blood antibacterial activity in many cases.

## DISCUSSION

The aim of this first-in-human phase 1 study was to provide an initial assessment of the safety, tolerability, pharmacokinetics, and pharmacodynamics of SAL200 in healthy men after the infusion of single ascending, intravenous doses (0.1, 0.3, 1, 3, and 10 mg/kg of body weight).

No serious AEs were observed. Most reported AEs were transient, self-limiting, and mild. No clinically significant values with respect to the findings of clinical chemistry, hematology, and coagulation analyses, urinalysis, vital signs, and physical examinations were observed. No notable trends in the ECG results were observed. Based on the acceptable safety and tolerability of SAL200 in humans described in this paper, we recommend that SAL200 be used an investigational drug in future efficacy studies in humans. Nevertheless, more intensive safety evaluations (e.g., trials of multiple ascending doses) are necessary to improve our understanding of the safety profile of the intravenous administration of SAL200.

SAL200 exhibited acceptable pharmacokinetic characteristics during and after intravenous infusion. One noteworthy point is the greater-than-dose-proportional increase in *C*_max_ and AUC observed in our study. Although a more extensive study is required, this phenomenon might be caused by the saturated clearance mechanism of SAL200. Given the molecular weight of rSAL-1, its renal clearance and distribution from the intravascular to the extravascular space should be minimal. Thus, we assume that the clearance of SAL200 is primarily explained by the proteolysis of SAL200 via plasma proteases and is partially explained by the degradation of SAL200 aggregate, which is degraded either by the proteasome or by the lysosome via autophagy.

For the pharmacodynamic characteristics of intravenously infused SAL200, blood antibacterial activity of greater than 0.078 μg/ml was detected in all blood samples collected from all active pharmaceutical ingredient-treated participants in cohorts 3 to 5 at 1 h postdosing. The blood antibacterial activity determined in this study guarantees that a dosing regimen of more than 1 mg/kg of SAL200 is a viable treatment option and might be effective in clinical practice on the basis of the following observations: (i) the minimum bactericidal concentration (MBC) of SAL200 was 0.078 μg/ml, as determined for a bacterial population of 1 × 10^6^ CFU/ml in a serum environment, and (ii) the value of TOD_50_, which is equivalent to a one-half-log drop in the initial concentration of viable bacteria, was less than 10 min (25). Therefore, the pharmacodynamic characteristics of intravenously infused SAL200 support the potential use of SAL200 as a new therapeutic drug.

Meanwhile, the blood antibacterial activity of SAL200 after intravenous infusion was significantly lower than the corresponding serum concentration of SAL200. This apparent discrepancy between the pharmacokinetic results (measuring immune-reactive rSAL-1) and the pharmacodynamic results (measuring biologically active rSAL-1) can be explained by certain proteolytic fragments of rSAL-1 immunoreactive with the monoclonal antibody used in the enzyme-linked immunosorbent assay (ELISA), which might have no or reduced antibacterial activity (data not shown). To understand the clinical significance of the pharmacodynamics of SAL200, the cause for this discrepancy between the pharmacokinetic and pharmacodynamic results needs further analysis.

To obtain additional valuable information, assessment of the time course of the development of immunogenicity should be included in the next human clinical trial. Although the data regarding immunogenicity on day 50 are important, they do not provide information about the time course of antidrug antibody development. The time course of immunogenicity is relevant information and would be helpful to establish repeated-dose treatment regimes.

SAL200, a new phage endolysin-based candidate drug used to treat staphylococcal infections, possesses several beneficial properties, including potent and rapid bacteriolytic effects and activity against drug-resistant bacterial strains and bacteria in biofilms (25). This rapid killing property makes SAL200 well suited to quickly reduce the bacterial burden in infected hosts. Furthermore, its activity against drug-resistant bacterial strains provides a potential solution for antibiotic resistance problems, and its activity against bacteria in biofilms provides an opportunity for therapeutic use against biofilm-associated infections, such as endocarditis. In addition, the possibility of the development of resistance to SAL200 is significantly lower than that to conventional antibiotics: we have not observed any mutant resistant to SAL200 during its development, and in the earlier resistance development experiments, even repeated exposure of an S. aureus strain to half of the MIC of SAL200 30 times failed to identify resistant mutants (data not shown). Unlike most antibiotics, SAL200 does not require bacterial metabolism or growth for activity, and it is bacteriolytic upon contact, which indicates that it is a potential therapeutic agent for recurrent infections related to antibiotic-tolerant bacterial strains.

The current study constitutes the first case report of an intravenously administered phage endolysin-based drug in humans. We anticipate that this work will provide incentives for developing clinical studies that investigate phage endolysin treatments to combat multidrug-resistant staphylococcal and other bacterial infections. This is especially important as the number of bacterial pathogens that are resistant to antibiotics continues to increase but the rates of discovery and the approval of new antibiotic therapeutics steadily decline.

## MATERIALS AND METHODS

### Ethics and regulatory approvals.

The Republic of Korea Ministry of Food and Drug Safety (MFDS) and the Institutional Review Board of Seoul National University Hospital (IRB no. H-1304-063-481) approved the protocol for this clinical study (protocol no. SAL200-1A). This study was registered with the Clinical Trials Registry (ClinicalTrials.gov identifier NCT01855048) and the Clinical Research Information Service (https://cris.nih.go.kr/cris/; identifier KCT0000968).

### Drug.

SAL200 was prepared in a formulation containing 18 mg/ml rSAL-1, 1.56 g/liter l-histidine (pH 6.0), 50 g/liter d-sorbitol, 1.47 g/liter CaCl_2_·2H_2_O, and 1 g/liter poloxamer 188 as previously described (24) in accordance with good manufacturing practice (GMP). The purity of rSAL-1 was greater than 95%, as confirmed via sodium dodecyl sulfate-polyacrylamide gel electrophoresis and size exclusion high-performance liquid chromatography. The levels of endotoxin, host cell DNA, and host cell protein in the test article were less than 1 endotoxin units/mg, less than 30 pg/mg, and less than 100 ng/mg, respectively. A formulation buffer (1.56 g/liter l-histidine [pH 6.0], 50 g/liter d-sorbitol, 1.47 g/liter CaCl_2_·2H_2_O, 1 g/liter poloxamer 188) was used as a placebo.

### Design.

This clinical study was conducted at a single center (Seoul National University Hospital, Seoul, Republic of Korea) from August 2013 to February 2014. The study's protocol was conducted in accordance with the Declaration of Helsinki. This randomized, double-blind, placebo-controlled study assessed the safety, tolerability, pharmacokinetics, and pharmacodynamics of single ascending intravenously infused doses of SAL200 in healthy men. The study consisted of a screening period (days −28 to −2), a run-in period (day −1), the treatment period (days 1 to 3), a follow-up visit (day 7 ± 1), and a poststudy visit (day 50 ± 7). An outline of the study is presented in Table 7. Five sequential dose cohorts (0.1, 0.3, 1, 3, and 10 mg/kg) were applied on the basis of a safety review until day 7 (follow-up visit).

**TABLE 7:** Outline of the study

Item	Screening (days −28 to −2)	Run-in period (day −1)	Treatment period			Follow-up visit (day 7 ± 1)	Poststudy visit (day 50 ± 7)
			Day 1	Day 2	Day 3		
Informed consent	•						
Skin hypersensitivity test		•					
Dosing			•				
Blood sampling for pharmacokinetic analysis			•	•			
Blood sampling for pharmacodynamic analysis			•				
Vital sign tests	•		•	•	•	•	•
Assessment of 12-lead ECG	•		•	•		•	•
Physical examination	•		•	•	•	•	•
Clinical laboratory tests	•			•		•	•
Urinalysis screening test	•						
Blood sampling for assay for SAL200 immunogenicity	•						•
Monitoring and verification of adverse events and concomitant medication	•	•	•	•	•	•	•

After fasting overnight, the participants received 100 ml of a medicinal product (either SAL200 or placebo) via intravenous infusion for approximately 1 h. The medicinal product was formulated using normal saline. The participants were allowed to drink water freely except between 1 h before and 1 h after dosing.

### Participants.

Clinical trial participants were recruited from among the members of the healthy adult male population of the Republic of Korea. A total of 36 healthy Korean male volunteers participated in this study after being fully informed of the study, its purpose, and the associated risks. The volunteers provided written informed consent to participate before enrollment. These participants were judged to be healthy on the basis of a physical examination, vital signs, a 12-lead electrocardiogram (ECG), and standard laboratory testing, including biochemical and hematological tests, as well as urinalysis. In addition, their suitability for this study was evaluated on the basis of a urine drug screening, an assay for SAL200 immunogenicity, skin hypersensitivity testing, and their medical history. A level of antibodies to rSAL-1 of less than 0.8 μg/ml was considered a negative result of the assay for SAL200 immunogenicity, and only participants who tested negative were included in this study. For skin hypersensitivity testing of the participants, approximately 100 μl of 0.05% SAL200 in physiological saline solution was introduced subcutaneously in the left arm and approximately 100 μl of 0.05% placebo in physiological saline solution was introduced subcutaneously in the right arm. A red, raised bump measuring more than 3 mm was considered a positive reaction. Participants testing positive were then excluded from this study.

Eligible participants were randomly assigned to receive either SAL200 or a matched placebo in a 3:1 design across one of five sequential dose cohorts (0.1, 0.3, 1, 3, and 10 mg/kg). Cohort 1 contained four participants (three active pharmaceutical ingredient-treated participants and one placebo-treated participant), whereas the other cohorts contained eight participants (six active pharmaceutical ingredient-treated participants and two placebo-treated participants in each cohort).

### Blood sampling.

Blood samples (10 ml) for pharmacokinetic analysis were collected before dosing and at 0.25, 0.5, 0.75, 1, 1.5, 2, 3, 4, 5, 6, 8, 10, 12, and 24 h after the start of intravenous dosing. Blood samples (1 ml) for pharmacodynamic analysis were collected at 1, 1.5, and 2 h after the start of intravenous dosing. All blood samples were allowed to stand for 30 min at room temperature and were centrifuged at 2,000 × *g* for 15 min to separate the serum. The serum samples were frozen at −20°C or below until analysis.

### Safety and tolerability evaluation.

Safety and tolerability were evaluated throughout the study via physical examination, vital sign testing, a 12-lead ECG assessment, and clinical laboratory testing. Physical examinations of the participants were conducted before dosing, at 24 and 48 h after dosing, at the 7-day follow-up visit, and at the 50-day poststudy visit. The testing of the vital signs blood pressure and pulse rate (PR) was conducted before dosing and at 0.5, 1, 2, 4, 8, 12, 24, and 48 h after dosing as well as at the 7-day follow-up visit and the 50-day poststudy visit. In addition, the testing of the vital sign body temperature was conducted at the 7-day follow-up and 50-day poststudy visits. A 12-lead ECG assessment (ventricular rate, PR interval, QRS duration, and QT/QTc interval) was conducted at 0.5, 1, 4, 8, and 24 h after dosing as well as at the 7-day follow-up visit and the 50-day poststudy visit. Clinical laboratory testing was conducted on day 2, at the 7-day follow-up visit, and at the 50-day poststudy visit; this testing included standard hematological analyses, coagulation analyses, serum biochemical assays, and urinalysis. A SAL200 immunogenicity assay was conducted using the sample obtained at the 50-day poststudy visit.

Safety and tolerability were also evaluated on the basis of adverse events (AEs). All AEs were recorded and classified using *Medical Dictionary for Regulatory Activities* (MedDRA)-preferred terms.

### Analyses.

To assess the humoral immune response to SAL200, the serum levels of antibodies against rSAL-1 were determined using a conventional bridging enzyme-linked immunosorbent assay (ELISA) with streptavidin-biotin detection. For the pharmacokinetic analysis, rSAL-1 levels in serum were determined using a validated double-antibody sandwich ELISA. Pharmacokinetic calculations were performed using the pharmacokinetic program Phoenix WinNonlin (version 6.3; Pharsight, Mountain View, CA, USA) via noncompartmental methods. Pharmacokinetic parameters, including the maximum drug concentration in serum (*C*_max_), the time to *C*_max_ (*T*_max_), the effective terminal half-life (*t*_1/2_), and the area under the serum concentration-time curve (AUC), were determined. Pharmacodynamics were measured with an *ex vivo* blood assay for determining blood antibacterial activity. Serum samples collected after intravenous dosing were used for the *ex vivo* blood assay. Detailed descriptions of the methods relating to immunogenicity assessment, determination of rSAL-1 levels in serum, pharmacokinetic analysis, and pharmacodynamic assays are provided in the supplemental material.

### Statistical analyses.

All demographic information and safety and pharmacokinetic data were summarized on the basis of appropriate criteria, such as dose group and treatment group, using descriptive statistics. Unless otherwise indicated, continuous variables were summarized using the following descriptive statistics: number of observations (*n*), mean, standard deviation (SD), minimum, median, and maximum. Categorical data were summarized using frequencies and percentages. Percentages within categories were based on the number of participants exposed to a treatment. Pharmacokinetic and pharmacodynamic analyses were performed to correlate the serum concentration of SAL200 with the blood antibacterial activity measured simultaneously for the same participants. All statistical analyses were performed using SPSS (version 21; IBM Corporation, Armonk, NY, USA) and SAS (version 9.3; SAS Institute, Cary, NC, USA) software.

## Supplementary Material

Supplemental material
