# Supplementary material for: Pharmacokinetics and Tolerance of the Phage Endolysin-Based Candidate Drug SAL200 after a Single Intravenous Administration among Healthy Volunteers
Source: Antimicrob Agents Chemother. 2017 May 24;61(6):e02629-16. doi: 10.1128/AAC.02629-16 (PMC5444177; doi:10.1128/AAC.02629-16)
Supplement: Supplemental material [file supp_61_6_e02629-16__index.html]

Pharmacokinetics and Tolerance of the Phage Endolysin-Based Candidate Drug SAL200 after a Single Intravenous Administration among Healthy Volunteers — Supplemental material 

# Pharmacokinetics and Tolerance of the Phage Endolysin-Based Candidate Drug SAL200 after a Single Intravenous Administration among Healthy Volunteers

## Supplemental material

- Supplemental file 1 -

  Supplemental methods and Table S1.

  PDF, 95K
