# Supplementary material for: Pharmacokinetics and Tolerance of the Phage Endolysin-Based Candidate Drug SAL200 after a Single Intravenous Administration among Healthy Volunteers
Source: Antimicrob Agents Chemother. 2017 May 24;61(6):e02629-16. doi: 10.1128/AAC.02629-16 (PMC5444177; doi:10.1128/AAC.02629-16)
Supplement: Supplemental material [file AAC.02629-16_zac006176213s1.pdf]

SUPPLEMENTAL METHODS

Immunogenicity assessment. To assess the humoral immune response to SAL200, the serum level of antibodies against rSAL-1 was determined using a conventional bridging ELISA with streptavidin-biotin detection. As an antibody standard, anti-rSAL-1 immunoglobulins (2 mg/ml) were prepared according to the conventional IgG purification method from immune sera obtained from dogs immunized with SAL200. The prepared anti-rSAL-1 immunoglobulins were confirmed via immunodepletion testing. Biotinylated rSAL-1 (1 mg/ml) was obtained from Younginfrontier (Seoul, Republic of Korea). The streptavidin-HRP solution (1 mg/ml) was obtained from Sigma-Aldrich (St. Louis, MO, USA). An ELISA starter kit was obtained from Koma Biotech (Seoul, Republic of Korea). ELISA was performed using a conventional method with modification. Briefly, SAL200 was diluted to 1 mg/ml in a formulation buffer (1.56 g/l L-histidine [pH 6.0], 50 g/l D-sorbitol, 1.47 g/l $CaCl_2 \cdot 2H_2O$, and 1 g/l poloxamer 188). The diluted SAL200 solution was further diluted to 1 μg/ml using the coating buffer (0.05 M sodium carbonate buffer, pH 9.6) provided in the ELISA starter kit. The prepared solution (100 μl) was added to each well of an ELISA plate and then covered with a plate sealer, after which the plate was incubated at 37°C for 1 h. The plate was subsequently washed three times by applying 250 μl of washing buffer (phosphate-buffered saline [PBS] containing 0.05% Tween-20) per well. For blocking, 200 μl of blocking buffer (PBS containing 0.05% Tween-20, 0.5% bovine serum albumin, and 20% fetal bovine serum) was added to each well to block the remaining binding sites on the plate. After the addition of the blocking buffer, the plate was incubated for 2 h at 37°C. Next, the wells were washed as described above. ELISA samples, including calibration standards, quality control standards, and test samples, were prepared during the blocking and washing

steps. Calibration standards, including anchor points (0.781, 1.563, 3.125, 6.25, 12.5, 25, 50, and 100 μg/ml) and quality control standards (2, 10, and 40 μg/ml), were prepared using the anti-rSAL-1 antibody standard and human pooled serum. The calibration standards, quality control standards, and test samples were diluted 1:20 in PBS, and the mixtures were briefly vortexed. The prepared ELISA samples (100 μl) were added to the wells of the plate and shaken for 1 min using an orbital shaker. The plate was subsequently incubated for 1 h at 23°C. After incubation, the wells were washed again as described above. A solution of 1:10,000 biotinylated rSAL-1 was prepared in a dilution buffer (PBS containing 0.05% Tween-20, 0.5% bovine serum albumin, and 10% fetal bovine serum). Then, 100 μl of the diluted biotinylated rSAL-1 solution was added to each well of the plate, and the plate was covered with a plate sealer. The plate was then incubated for 1 h at 23°C, and the wells were re-washed. A 1:10,000 streptavidin-HRP solution was prepared in a dilution buffer (PBS containing 0.05% Tween-20 and 0.5% bovine serum albumin). The diluted streptavidin-HRP solution (100 μl) was added to each well, and the plate was covered with a plate sealer. The plate was incubated for 1 h at 23°C. After incubation, the wells were re-washed. Next, 100 μl of the provided tetramethylbenzidine (TMB) substrate solution was added to each well, and the plate was incubated for no longer than 30 min at room temperature in the dark. The reaction was stopped by the addition of 100 μl of stop solution, and the OD_{450} was recorded. The ELISA was effective in an analysis range of 1.563-50 μg/ml, and the within-run accuracy, within-run precision, between-run accuracy, and between-run precision values were 95.49-107.5%, 1.770-4.919%, 94.50-110.0%, and 2.342-7.012%, respectively.

Determination of rSAL-1 levels in serum. For the pharmacokinetic analyses, the rSAL-1 levels in serum were determined using a validated double antibody sandwich ELISA. A monoclonal anti-rSAL-1 antibody and a monoclonal anti-rSAL-1 antibody conjugated to

HRP were obtained from Younginfrontier (Seoul, Republic of Korea). An ELISA starter kit was obtained from Koma Biotech (Seoul, Republic of Korea). ELISA was performed according to a conventional method with modification. Briefly, 1:400 dilutions of a 2-mg/ml solution of monoclonal anti-rSAL-1 antibody (capture antibody) were prepared with a coating buffer (0.05 M sodium carbonate buffer, pH 9.6). The diluted capture antibody solution (100 μl) was added to each well of the ELISA plate and then covered with a plate sealer, after which the plate was incubated overnight at 4°C. The plate was subsequently washed five times by applying 250 μl of a washing buffer (Tris-buffered saline [TBS] containing 0.05% Tween-20) per well. For blocking, 200 μl of blocking buffer I (Applichem, Darmstadt, Germany) was added to each well to block the remaining binding sites on the plate. After the addition of the blocking buffer, the plate was incubated for 1 h at 37°C. Next, the wells were washed as described above. ELISA samples, including calibration standards, quality control standards, and test samples, were prepared during the blocking and washing steps. Calibration standards, including anchor point (0.008, 0.016, 0.031, 0.063, 0.125, 0.250, and 0.500 μg/ml) and quality control standards (0.048, 0.095, and 0.350 μg/ml), were prepared using SAL200 and human pooled serum. The calibration standards, quality control standards, and test samples were then diluted 1:10 in TBS containing 5.5 mM $CaCl_2$, and the mixtures were briefly vortexed. The prepared ELISA samples (100 μl) were added to the wells of the plate and shaken for 1 min using an orbital shaker. The plate was subsequently incubated for 3 h at 25°C. After incubation, the wells were re-washed as described above. A 2.5-mg/ml solution of monoclonal anti-rSAL-1 antibody conjugated to HRP (detection antibody) was diluted 1:5,000 in TBS. After re-washing the plate, 100 μl of the diluted detection antibody solution was added to each well of the plate, and the plate was covered with a plate sealer. The plate was then incubated for 2 h at 25°C, and the wells were re-

washed. Next, 100 μl of the TMB substrate solution was added to each well, and the plate was incubated for no longer than 30 min at room temperature in the dark. The reaction was stopped by the addition of 100 μl of stop solution, and the OD_{450} was recorded. This ELISA method was effective in an analysis range of 0.016-0.500 μg/ml, and the within-run accuracy, within-run precision, between-run accuracy, and between-run precision values were 96.88-116.7%, 2.259-15.67%, 93.92-103.4%, and 8.670-12.15%, respectively.

Pharmacokinetic analysis. Pharmacokinetic calculations were performed using the pharmacokinetic program Phoenix™ WinNonlin® version 6.3 (Pharsight, Mountain View, CA, USA) with non-compartmental methods. The C_{max} and t_{max} values were obtained directly from the serum concentration-time profiles. The $t_{1/2}$ value over the sampling period was calculated as the mean residence time from the time of dosing to the time of the last quantifiable concentration (MRT_{last}): $MRT_{last} \times 0.693$. MRT_{last} was calculated as $AUMC_{last} / AUC_{last}$, where $AUMC_{last}$ denotes the area under the first moment curve from time zero to the time at which the last sample with a measurable concentration was obtained. The area under the serum concentration-time curve from time zero to the time at which the last sample with a measurable concentration (AUC_{last}) was calculated using the linear trapezoidal rule for the ascending region of the concentration-time curve and the log-linear trapezoidal rule for the descending region of the concentration-time curve. Data below the LLOQ were excluded from the AUC_{last} calculation. The AUC from time zero to infinity (AUC_{inf}) was estimated as $AUC_{last} + C_{last} / K_{el}$, where C_{last} denotes the concentration in the last serum sample at which a measurable concentration was obtained. The total body clearance (CL) was calculated as the dose / AUC_{inf}. All data were expressed as the means ± SDs.

Pharmacodynamic assay. Pharmacodynamics were measured using an *ex vivo* blood assay for determining blood antibacterial activity. Serum samples collected after

intravenous dosing were used in the *ex vivo* blood assay. Briefly, 20 μl of *S. aureus* glycerol stock was inoculated into a 50-ml conical tube containing 20 ml of tryptic soy broth (TSB; 17 g/l casein digest, 3 g/l soybean digest, 2.5 g/l dextrose, 5 g/l NaCl, and 2.5 g/l dipotassium phosphate) and incubated overnight at 37°C. Next, 20 μl of the overnight bacterial culture was added to a 50-ml conical tube containing 20 ml of TSB medium. The conical tube was incubated at 37°C until the OD_{600} reached 1.8-2.0, at which time 2 ml of culture was distributed evenly across the surface of a tryptic soy agar (TSA; 15 g/l casein digest, 5 g/l soybean digest, 5 g/l NaCl, and 15 g/l agar) plate, and the excess broth was removed after standing for 5 min. The inoculated plate was then allowed to dry in a laminar flow hood for approximately 30 min with the lid ajar. After drying, the plate was incubated overnight at 37°C. Standards (0, 0.05, 0.1, 0.2, 0.4, 0.6, 0.8, and 1 μg/ml) were subsequently prepared by spiking SAL200 into human pooled serum containing 10 mM $CaCl_2$. Test samples were prepared by adding a 1% (v/v) volume of 1 M $CaCl_2$ to the serum samples collected from each participant. Then, 10-μl aliquots of standard and test samples were spotted onto the plate. The plate was allowed to dry in a laminar flow hood for approximately 10 min with the lid ajar. The plate was incubated overnight at 37°C. Following incubation, the pharmacodynamics were accessed via a qualitative comparison of the clear zones (i.e., lysis halos) formed after spotting the test samples onto the lawn of *S. aureus* with those formed after spotting the standards.

SUPPLEMENTAL TABLE

Table S1. Adverse events after a single intravenous dose among healthy male volunteers (cohort 5, individual)

Participant identification code (dose, mg/kg; A/P[a])	Dosing date	Observed adverse event (number of adverse events)	Observed time				Comment (number of adverse events)
			Day 1[b]	Day 2	Day 3	≥ Day 7	
R501 (10; A)	Nov 12, 2013	Rigors (1)	●				Related (1)
		Dizziness (1)	●				Related (1)
		Myalgia (2)	●			●	Related (2)
		Headache (1)	●				Related (1)
		Abdominal pain (1)		●			Related (1)
		Fatigue (1)		●			Related (1)
		Pharyngitis (1)			●		Related (1)
		Rhinitis (1)				●	Related (1)
R502 (10; A)	Nov 12, 2013	Nausea (1)	●				Related (1)
		Dizziness (1)	●				Related (1)
		Rigors (1)	●				Related (1)
		Fatigue (1)	●				Related (1)
		Myalgia (1)	●				Related (1)
		Hypotension (1)	●				Related (1)
R503 (10; A)	Nov 12, 2013	Rigors (1)	●				Related (1)
		Headache (1)	●				Related (1)
		Back pain (1)	●				Related (1)

		Nausea (1)	●			Related (1)
		Fatigue (1)	●			Related (1)
		Dyspepsia (1)	●			Related (1)
R504 (10; P)	Nov 19, 2013	None				
R505 (10; A)	Nov 19, 2013	Rigors (1)	●			Related (1)
		Headache (1)	●			Related (1)
R506 (10; A)	Nov 26, 2013	Rhinitis (2)	●	●		Related (1)/ Unrelated (1)
R507 (10; P)	Nov 26, 2013	Headache (3)	●		●/●	Related (2)/ Unrelated (1)
R508 (10; A)	Nov 26, 2013	Fever (1)	●			Related (1)
		Rhinitis (1)			●	Unrelated (1)
		Coughing (1)			●	Unrelated (1)

[a]A, active; P, placebo

[b]Dosing day
